# Comparing Three Different Extraction Techniques on Essential Oil Profiles of Cultivated and Wild Lotus (*Nelumbo nucifera*) Flower

**DOI:** 10.3390/life10090209

**Published:** 2020-09-16

**Authors:** Chun-Yun Zhang, Mingquan Guo

**Affiliations:** 1 CAS Key Laboratory of Plant Germplasm Enhancement and Specialty Agriculture, Wuhan Botanical Garden, Chinese Academy of Sciences, Wuhan 430074, China; cyzhang@wbgcas.cn; 2 Sino-African Joint Research Center, Chinese Academy of Sciences, Wuhan 430074, China; 3 Innovation Academy for Drug Discovery and Development, Chinese Academy of Sciences, Shanghai 201203, China

**Keywords:** extraction technique, essential oil, *N. nucifera* flower, GC-MS

## Abstract

Essential oil components of *Nelumbo nucifera* flowers from cultivated and wild lotus samples were analyzed and compared using three different extraction techniques, i.e., headspace extraction (HE), steam distillation (SD) and solvent extraction (SE), coupled with GC-MS. Forty-two peaks in the GC-MS analysis were identified as essential oil components extracted by the three methods from *N. nucifera* flower. The major essential oil components extracted by SD method were found to be *Z*,*Z*-10,12-hexadecadienal and *E*-14-hexadecenal with relative contents of 16.3% and 16.7%, respectively, which is different from that of SE method, i.e., n-hexadecanoic acid and *Z*,*Z*-9,12-octadecadienoic acid accounting for 25.8% and 26.8%, respectively. HE method demonstrated a possibility to be used as an in situ and simplest method for extracting the essential oil components from raw materials. By adding a small amount of glycerinum onto the surface of the air-dried flower sample as a solvent trap in the HE method, the volatility of the essential oil components was found to increase by two times for the first time, which could be further utilized to improve the extraction efficiency and the recovery of the essential oil components from other materials for more applications. In addition, the comparison of essential oil components between cultivated and wild samples showed that they differed only in the chemical contents but not in chemical components. This will be a comprehensive report on the chemical information of the essential oil components of *N. nucifera* flower and provide guidance for its further exploration as high value-added products in the food and healthcare industries.

## 1. Introduction

*Nelumbo nucifera*, commonly known as lotus, is an aquatic perennial plant, which has been cultivated in most provinces of China, and even across many parts of the world [1]. It has mainly been used as aquatic vegetable, and almost all parts of *N. nucifera* have been found to be very useful in either traditional herbal medicines or healthcare foods [2,3,4,5]. The flower of *N. nucifera* was primarily used for personal health care products, such as body lotions and bath soaps, or for producing scented substances in green tea [6]. Its medicinal value is rarely reported but considered to be associated with aromatherapy, e.g., the treatment of respiratory problems. In vitro studies with human melanocytes demonstrated that the essential oil extract of lotus flower has effects of increasing the melanogenesis, representing a potential use for photoprotection [7]. To further explore the healthcare functions of the essential oil of lotus flower, the study of its chemical information and the effect of extraction method on the chemical composition, presented in essential oil of *N. nucifera*, is of paramount importance for the quality control of essential oil derived products from this plant material.

In order to analyze the chemical components of essential oil in *N. nucifera*, the sample pretreatment is a pre-requisite and mandatory step prior to the analysis. For the essential oil components in plants, enfleurage and cold pressing are the traditional methods used in the applications [8]. However, these methods are more suitable for the plant samples rich in essential oils. For the samples with small amount of essential oil, steam distillation and solvent extraction were the commonly used techniques. Steam distillation has been widely used in industrial production of essential oil because the use of water is environmental-friendly and economically [9,10,11]. Since some components in essential oils are too delicate and thus easily denatured at high-temperature steam distillation, liquid-extraction using organic solvent (e.g., hexane), has also been used for extracting essential oils from plants [12,13]. Although solvent extraction can obtain relatively more essential oils, the procedures for sample concentrating (by solvent evaporation) and re-dissolving are time-consuming and easy to introduce other chemical impurities, such as non-volatile components co-existing and solvent remaining during the solvent extraction step.

Headspace based extraction techniques, e.g., static or dynamic headspace, solid phase micro-extraction (SPME, using a phase coated fused-silica fiber as the adsorption medium), have been proposed as simpler techniques for extracting essential oil components from plant species [14,15,16]. The major advantage of headspace-based extraction techniques is that the analyte(s) released to headspace or a phase coated fused-silica fiber can be immediately measured by gas chromatography coupled with FID or MS detector. The most commonly used technique in headspace-based extraction is SPME. However, the poor repeatability, caused by the loss of a phase coated material on fused-silica fiber, is a major problem in the quantitative analysis [17,18]. Static headspace (SH) technique, usually operating with an automatic headspace sampler, has proven itself in numerous applications for analyzing volatile species in the presence of non-volatile interferences [19]. Coupled with GC or GC-MS, SH can be used to simultaneously determine multiple volatile species in a very complex sample. Since the essential oil components in *N. nucifera* flowers are gradually released into the headspace at an elevated temperature, and their concentrations can be accumulated in the headspace with the time increasing. Thus, an in situ and automatic extraction and measurement of essential oil from *N. nucifera* flowers can be achieved. However, it is not available for the low-volatile components.

The essential oil components of lotus flower were typically reported for wild-type materials with only one extraction method [5]. To facilitate large-scale use of this plant material, it is important to include the cultivated material sources and multiple extraction methods to account for potential variation in the essential oil components. Therefore, the main focuses of this work were on the analysis of the essential oil components of *N. nucifera* flowers, including components extraction, separation and identification, and the comparison of the essential oil components extracted by different techniques. Based on this, the essential oil components of two *N. nucifera* flower samples from different growing environments, i.e., wild-type and cultivated samples, were compared. This will provide basic chemical information for the further exploitation on the essential oil extracted from *N. nucifera* flower.

## 2. Experimental

### 2.1. Chemicals and Materials

All chemicals, including n-hexane, glycerinum and sodium chloride used in the experiment, were of analytical grade and purchased from commercial sources without further purification. Water for steam distillation solution preparation was prepared daily with a Millli-Q purification (Millipore, Bedford, MA, USA).

The cultivated fresh *N. nucifera* flowers were collected from Wuhan botanical garden in August 2015. The wild fresh *N. nucifera* flowers were collected from Wuhan, Hubei province in August 2015. The authentication and identification of the specimens were assisted by Prof. Guangwan Hu, a senior taxonomist of Key Laboratory of Plant Germplasm Enhancement and Agriculture Specialty (Wuhan Botanical Garden, Wuhan, China), Chinese Academy of Sciences. Some of the two fresh samples (more than 200 g) were preserved in the freezer at a temperature of −40 °C for steam distillation. The rest of two samples were air-dried and grinded to powers which can pass through the sieves with 40 meshes for headspace extraction and solvent extraction.

### 2.2. Procedures for Sample Preparation

Headspace Extraction (HE). One gram (g) of powdered sample (wild sample) and 0.5 g of pure glycerinum were weighed and placed into a headspace vial. Before the sample vial was sealed with a PTFE/silicone septum and an aluminum cap, the sample powder and glycerinum were completely mixed with a glass rod. Headspace extraction, followed by GC-MS measurement, was performed on the sample vial with the automatic headspace sampler.

Steam Distillation (SD). Two hundred grams of fresh *N. nucifera* flower (wild and cultivated sample) and 1600 g of 3% (*w/w*) sodium chloride solution was weighed and placed into a 2000 mL round flask. The flask was heated to boiling by an electric heater for 4 h after being slightly shaken and standing for 12 h in room temperature. For the isolation of the essential oils, a Clevenger apparatus was used. Finally, the essential oils were stored in a sealed vial at 4 °C for measurement.

Solvent Extraction (SE). Five grams of air-dried flower powders (wild sample) and 50 g of n-hexane was weighed and placed into a bottle. The bottle was then placed into the ultrasonic cleaning for extraction process. The ultrasound assisted solvent extraction was carried out under the following experimental conditions: temperature = 40 °C; time = 40 min; solid to solvent ratio = 1:10 (*w*/*w*); sonication frequency = 40 KHz. Three replicates were performed on each sample, and the extracts were combined and filtered with 0.45 μm membrane using a vacuum pump. The filtrate was evaporated to roughly 1 mL and stored in a sealed vial at 4 °C for measurement.

### 2.3. Apparatus and Operation Conditions

An automated headspace sampler (Agilent 7697A, Santa Clara, CA, USA) equipped with a sample loop volume of 1 mL, a GC system (Agilent GC 7890A, Santa Clara, CA, USA) equipped with HP-5 capillary column, and MS system (Agilent 5975C, Santa Clara, CA, USA), were used for the analysis of the essential oil components from *N. nucifera* flowers. The headspace operating conditions were as follows: equilibration time = 60 min; equilibration temperature = 150 °C; pressing time = 0.5 min; extracting time = 0.2 min; injecting time = 0.5 min. GC operating conditions were as follows: The carrier gas was helium, at a flow rate of 1 mL/min; the column temperature program of GC was initially set at 120 °C for 3 min and gradually increased to 200 °C at 4 °C /min, then kept there for 10 min before gradually increased to 260 °C at 12 °C /min, and then kept there for 10 min. For GC-MS measurements, an electron ionization system was used with ionization energy at 70 eV.

## 3. Results and Discussion

### 3.1. Chromatogram in the GC-MS Analysis of the Extracts

**Figure 1:**
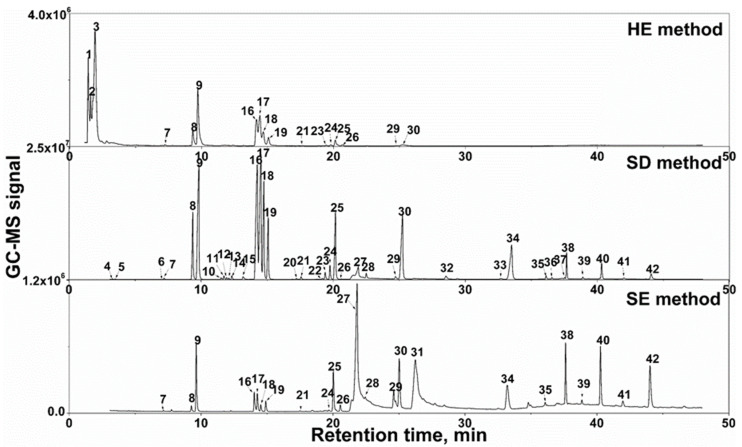
Chromatogram in the GC-MS analysis of the components of essential oil from *N. nucifera* flowers using three extraction techniques: HE method, SD method, and SE method.

The extracts of wild *N. nucifera* flowers using the three techniques, i.e., HE, SD and SE, were analyzed by GC-MS under the same operating conditions. Figure 1 shows the chromatogram of essential oil components of *N. nucifera* flower extracted by HE, SD and SE, respectively. It was observed that the essential oil components of the three methods can be well separated and measured by GC-MS under the given operating conditions, indicating that the optimized operating conditions can be used to analyze the essential oil components of extracts obtained by the three techniques. Notably, to avoid the chemical change in the headspace extraction caused by the elevated temperature and oxygen in the headspace, a small amount of glycerinum was added to the sample powder to produce a solvent membrane on the surface of the powder.

### 3.2. Identification of the Essential Oil Components

Forty-two peaks in Figure 1 were identified as essential oil components by comparing their mass fragmentation pattern with those stored in the NIST database using NIST 11. The results were listed in Table 1, in which the essential components were semi-quantified by relative peak areas of the total ion chromatography from the MS signals. It was found that the chemicals in essential oil from *N. nucifera* flower were the alkene aldehydes and alcohols, n-alkenes and n-alkanes, which were also reported on the essential oil components from other aromatic plant species, such as *Osmanthus fragrans*, *Thymus vulgaris* and *Lavandula angustifolia* [20,21]. Among them, the terpene aldehydes and alcohols were reported as the common chemicals with promising bioactivities [22]. Clearly, different chemical information, including composition and contents, was obtained among the three extraction techniques, which will be discussed below.

### 3.3. Comparison of the Essential Oil Components Extracted by Three Techniques

**Figure 2:**
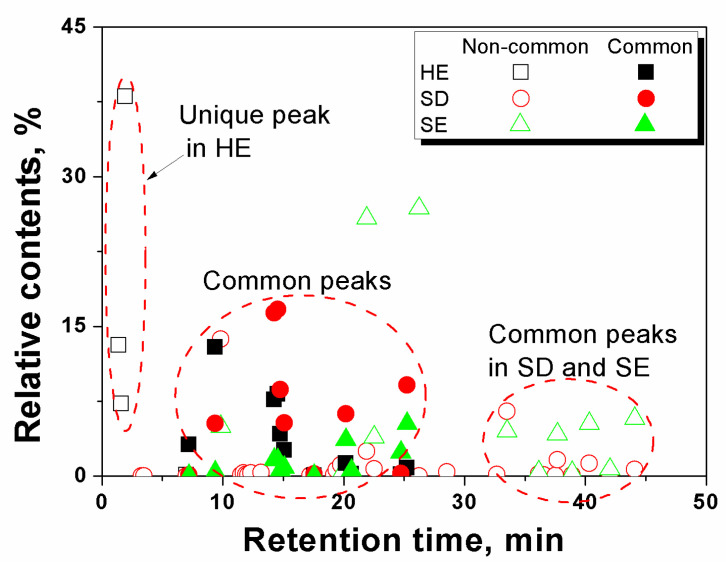
Common and unique components of essential oil extracted by the three techniques (HE, SD and SE) from *N. nucifera* flower.

Qualitative comparison. The common and unique components of essential oil among the three extraction techniques were summarized in Figure 2. It can be seen that 11 peaks were the common components among the three extraction techniques, which were mostly located in the range of moderate volatility. However, there exist great differences in essential oil components among the different extraction techniques. Up to 14 peaks were only found in SD method and SE method, while two were only associated with HE method and SD method, and no common component was found between HE method and SE method except the 11 common components among the three extraction techniques. Taken together, our results suggests the SD and SE used as the conventional methods can obtain more similar results in essential oil composition, and the HE method is preferable for components with relatively higher volatility. It is not surprising that the three methods resulted in different essential oil profiles in the extraction. Generally, the SE method is driven by the solid–liquid partitioning between sample matrix and the extraction solvent (n-hexane) and targeting relatively non-polar compounds, whereas HE method is driven by the solid–vapor partitioning between sample matrix and headspace and relies on the volatility of the analytes, whereas SD method involves two steps of partitioning, i.e., solid–liquid portioning between sample matrix and water and liquid–vapor partitioning between water and headspace. Given sufficient extraction time, the equilibrium states of the extractions eventually are determined by these partitioning coefficients between phases.

Quantitative comparison. From Figure 2, the quantitative differences of the essential oils extracted by the three methods can also be observed from the relative contents. In the HE method, acetic acid was found to be with the largest relative content of 38.1% more likely due to its high volatility. While in SD method, two olefine aldehydes were found to be the major components of essential oil, which were *Z*,*Z*-10,12-hexadecadienal and E-14-hexadecenal accounting for 16.3% and 16.7%, respectively. However, in the SE method, the main components of essential oil were two olefine acids, i.e., n-hexadecanoic acid and *Z*,*Z*-9,12-octadecadienoic acid accounting for 25.8% and 26.8%, respectively. It indicates that the components responded quite differently in the contents extracted by different techniques.

**Figure 3:**
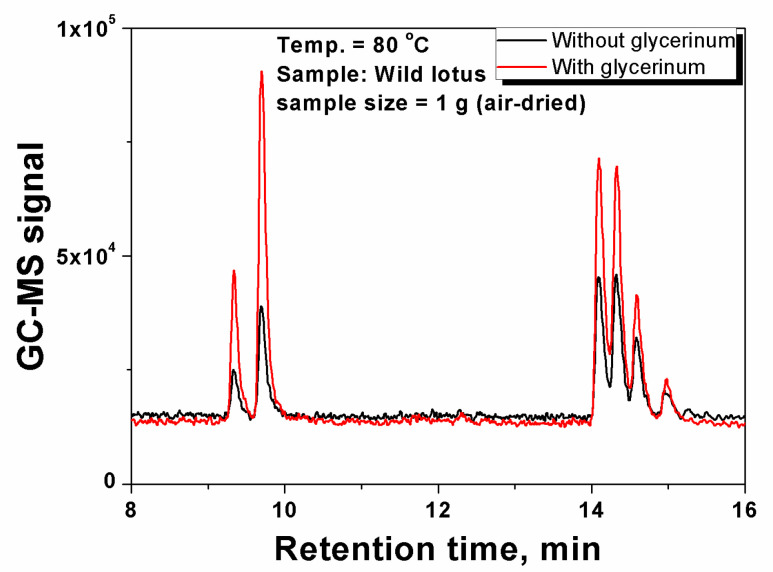
Effect of glycerinum on the detection sensitivity of volatile species in headspace analysis.

Selection of extraction techniques. Based on the above observations, it was likely that SD and SE used as the conventional extraction techniques can obtain similar results in essential oil composition but quite different in their contents. Therefore, the extraction techniques can be selected according to the targeted components. For example, SD is preferred for the enrichment of olefine aldehydes, and olefine acids are more accessible for SE. It is quite promising to develop HE method as an in situ and simplest method for the extraction of the essential oils, but it is probably limited to the components with higher volatility. Overall, the SE and SD methods are more suitable for achieving broader coverage of the essential oil components for both analytical and production purpose, whereas the HE method is preferable for targeted analysis of highly volatile components. Furthermore, some measures can be taken to enhance the volatility of targeted components to achieve a better coverage. In this work, glycerinum was used as a high-boiling-point solvent trap that scattered on the surface of the sample powders to enhance the driving force of gas phase partition of the volatile species. The results were shown in Figure 3. It can be seen for the first time that more than twice higher GC-MS signals were obtained in the sample with glycerinum than that without glycerinum, indicating that a high-boiling-point solvent can be used to improve the extraction efficiency of the tested components and thus increase the detection sensitivity.

### 3.4. Comparison of the Essential Oil Components between Wild and Cultivated Samples

It was reported that components of the essential oil from a plant not only depend on the metabolic state of the source tissues but also may be integrated with the stress factors from the growing environment [23,24]. The components of essential oil from lotus flower samples with different growing environments, i.e., the cultivated and wild lotus, were investigated using the steam distillation extraction coupled with GC-MS. This extraction method provides the highest number of essential oil components and facilitates a more comprehensive comparison (Table 1). The qualitative and quantitative information of the essential oil components from cultivated and wild lotus flower is summarized in Table 2. It was observed that almost the same essential oil components were detected in both *N. nucifera* flower samples.

**Figure 4:**
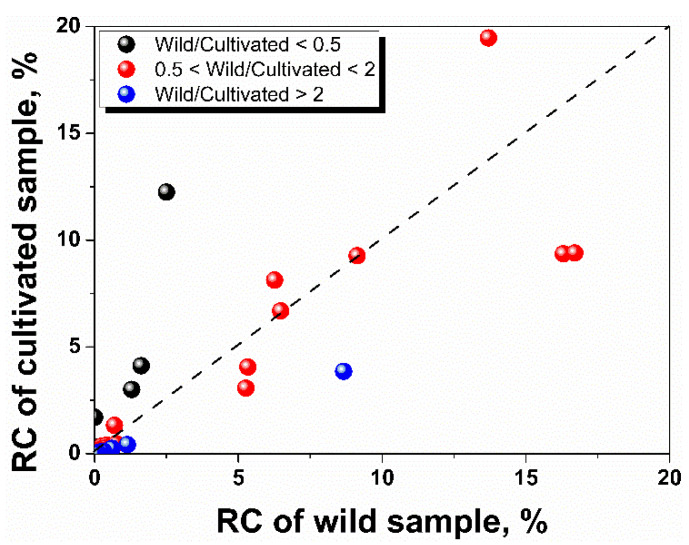
Differences of relative contents (RC) of essential oil from cultivated and wild lotus flowers.

Differences were found on the relative contents (RC) of essential oil components (Figure 4). Seventeen out of the 42 peaks were different by more than two times in their RC between wild and cultivated samples, including seven peaks with RC ratio (wild/cultivated) smaller than 0.5 (Peak **6**, **27**, **31**, **38**, **39** and **40**) and ten peaks with this ratio larger than 2 (Peak **4**, **11**, **13**, **18**, **20**, **22**, **23**, **24**, **33** and **36**). Particularly, four major components (with relative contents larger than 10 % in either wild or cultivated sample) showed significant differences in RC between the two samples, which are pentadcane and n-hexadecanoic acid with respective RC of 19.4% and 12.2% in cultivated sample and 13.7% and 2.50% in wild sample, and *Z*,*Z*-10,12-hexadecadienal and *E*-14-hexadecenal with respective RC of 9.36% and 9.40% in cultivated sample and 16.3% and 16.7% in wild sample. It is not surprising that the wild sample and the cultivated sample have different RC in their essential oil component because the overall exposure during growing may play an important role in the formation of these secondary metabolites. Both wild and cultivated *N. nucifera* are the source of this plant material in the application of essential oil-based products. However, our results provide basic chemical information for the quality assessment of essential oil products derived from lotus flower and suggest the differences in the quantities of essential oil components from different source needs to be considered.

## 4. Conclusions

The essential oil components of *N. nucifera* flower have been analyzed and compared using three different extraction techniques coupled with GC-MS. Steam distillation and solvent extraction techniques used as the conventional methods for the extraction of essential oil from lotus flowers can obtain similar components but different contents. Although headspace extraction is only favorable to the high volatile components of essential oil, it could be promising to develop an in situ and simpler method for analyzing essential oil in plant species when measures are taken to increase the driving force of gas phase partition of the components, e.g., by adding glycerinum onto the sample. *N. nucifera* flowers from different growing environments, i.e., cultivated and wild samples, showed difference in relative contents of essential oil but not in chemical components. This study provided the chemical extraction and identification information on essential oil from *N. nucifera* flower, which is of great importance for its further exploitation as high value-added products in the agricultural and healthcare industries.

## Figures and Tables

**Table 1:** Identification and comparison of the components of essential oil from wild *N. nucifera* flower using three extraction methods.

Peak Number	Retention Time, Min	Components ^a^	Relative Contents, %			Chemical Class
			HE	SD	SE	
7	7.188	tetradecane	3.16	0.0999	0.0696	Alkanes
9	9.832	pentadecane	-	13.7	4.98	
14	12.319	hexadecane	-	0.309	-	
19	15.078	heptadecane	2.61	5.33	0.879	
21	17.566	octadecane	0.0718	0.242	0.0729	
25	20.171	nonadecane	1.26	6.26	3.63	
28	22.505	eicosane	-	0.749	3.89	
30	25.248	heneicosane	0.822	9.13	5.27	
32	28.541	docosane	-	0.426	-	
34	33.507	tricosane	-	6.47	4.51	
35	36.121	trtracosane	-	0.248	0.405	
38	37.672	pentacocane	-	1.62	4.21	
39	38.899	hexacosane	-	0.127	0.367	
40	40.324	heptacosane	-	1.29	5.29	
41	42.013	octacosane	-	0.106	0.691	
42	44.079	noncosane	-	0.690	5.76	
6	7.000	7-tetradecene	0.128	0.00677	-	Alkenes
8	9.368	1-pentadecene	12.9	5.26	0.410	
10	11.508	*E*-1,9-hexadecadiene	-	0.107	-	
11	11.725	*Z*-7-hexadecene	-	0.315	-	
12	11.931	*Z*-3-hexadecene	-	0.151	-	
13	12.094	*Z*-8-hexadecene	-	0.114	-	
18	14.752	8-heptadecene	4.24	8.66	0.593	
20	17.213	*E*-5octadecene	-	0.0895	-	
23	19.390	*Z*-5-nonadecene	0.110	0.605	-	
24	19.753	1-nonadecene	-	1.14	0.0973	
29	24.719	10-heneicosene	0.191	0.267	2.40	
33	32.68	Z-9-tricosene	-	0.169	-	
36	36.561	1,12-docosadiene	-	0.113	-	
37	37.526	*Z*-12-pentacosene	-	0.0658	-	
4	3.286	terpinen-4-ol	-	0.0328	-	Alcohols
5	3.471	α-terpineol	-	0.0414	-	
15	13.17	γ-eudesmol	-	0.388	-	
1	1.406	furfural	13.1	-	-	Aldehydes
16	14.249	*Z*,*Z*-10,12-hexadecadienal	7.64	16.3	1.66	
17	14.534	*E*-14-hexadecenal	8.21	16.7	1.61	
22	19.180	*Z*-9,17-octadecadienal	-	0.101	-	
3	1.923	acetic acid	38.1	-	-	Acids
27	21.898	n-hexadecanoic acid	-	2.50	25.8	
31	26.229	*Z*,*Z*-9,12-octadecadienoic acid	-	-	26.8	
26	20.625	hexadecanoic acid, methyl ester	0.209	0.0311	0.541	Esters
2	1.587	unidentified	7.27	-	-	-

^a^ A peak was included as identified when the spectrum match score is over 80% using NIST 11 database.

**Table 2:** Comparison of the relative contents of the components in essential oil extracted by SD method between wild and cultivated samples ^a^.

Peak Number	Retention Time, Min	Components	Relative Contents ^b^, %	
			Cultivated	Wild
4	3.286	terpinen-4-ol	-	0.0328
5	3.471	α-terpineol	0.311	0.0414
6	7.000	7-tetradecene	0.145	0.00677
7	7.188	tetradecane	0.154	0.0999
8	9.368	1-pentadecene	3.07	5.26
9	9.832	Pentadecane	19.47	13.7
10	11.508	*E*-1,9-hexadecadiene	0.0561	0.107
11	11.725	*Z*-7-hexadecene	0.118	0.315
12	11.931	*Z*-3-hexadecene	0.0807	0.151
13	12.094	*Z*-8-hexadecene	0.0356	0.114
14	12.319	hexadecane	0.244	0.309
15	13.17	γ-eudesmol	0.265	0.388
16	14.249	*Z*,*Z*-10,12-hexadecadienal	9.36	16.3
17	14.534	*E*-14-hexadecenal	9.40	16.7
18	14.752	8-heptadecene	3.86	8.66
19	15.078	heptadecane	4.06	5.33
20	17.213	*E*-5octadecene	0.0231	0.0895
21	17.566	Octadecane	0.228	0.242
22	19.180	*Z*-9,17-octadecadienal	0.0232	0.101
23	19.390	*Z*-5-nonadecene	0.251	0.605
24	19.753	1-nonadecene	0.426	1.14
25	20.171	nonadecane	8.13	6.26
26	20.625	hexadecanoic acid, methyl ester	0.0415	0.0311
27	21.898	n-hexadecanoic acid	12.25	2.50
28	22.505	eicosane	0.473	0.749
29	24.719	10-heneicosene	0.149	0.267
30	25.248	heneicosane	9.27	9.13
31	26.229	*Z*,*Z*-9,12-octadecadienoic acid	1.71	-
32	28.541	docosane	0.406	0.426
33	32.68	*Z*-9-tricosene	0.0775	0.169
34	33.507	tricosane	6.688	6.47
35	36.121	trtracosane	0.356695	0.248
36	36.561	1,12-docosadiene	0.020671	0.113
37	37.526	*Z*-12-pentacosene	0.0569	0.0658
38	37.672	pentacocane	4.12	1.62
39	38.899	hexacosane	0.276	0.127
40	40.324	heptacosane	3.01	1.29
41	42.013	octacosane	0.203	0.106
42	44.079	noncosane	1.33	0.690

^a^ The essential oil was extracted with SD method. ^b^ The relative contents were calculated as analyte peak area/total peak area × 100.
